# Establishment of an indicator framework for the transmission risk of the mountain-type zoonotic visceral leishmaniasis based on the Delphi-entropy weight method

**DOI:** 10.1186/s40249-022-01045-0

**Published:** 2022-12-08

**Authors:** Zhuowei Luo, Zhengbin Zhou, Yuwan Hao, Jiaxin Feng, Yanfeng Gong, Yuanyuan Li, Yun Huang, Yi Zhang, Shizhu Li

**Affiliations:** National Institute of Parasitic Diseases, Chinese Center for Disease Control and Prevention (Chinese Center for Tropical Diseases Research); NHC Key Laboratory of Parasite and Vector Biology; WHO Collaborating Centre for Tropical Diseases, National Center for International Research On Tropical Diseases, Shanghai, 200025 China

**Keywords:** Mountain-type zoonotic visceral leishmaniasis, Transmission risk, Indicator framework, Delphi, Entropy weight, China

## Abstract

**Background:**

Visceral leishmaniasis (VL) is one of the most important neglected tropical diseases. Although VL was controlled in several regions of China during the last century, the mountain-type zoonotic visceral leishmaniasis (MT-ZVL) has reemerged in the hilly areas of China in recent decades. The purpose of this study was to construct an indicator framework for assessing the risk of the MT-ZVL in China, and to provide guidance for preventing disease.

**Methods:**

Based on a literature review and expert interview, a 3-level indicator framework was initially established in November 2021, and 28 experts were selected to perform two rounds of consultation using the Delphi method. The comprehensive weight of the tertiary indicators was determined by the Delphi and the entropy weight methods.

**Results:**

Two rounds of Delphi consultation were conducted. Four primary indicators, 11 secondary indicators, and 35 tertiary indicators were identified. The Delphi-entropy weight method was performed to calculate the comprehensive weight of the tertiary indicators. The normalized weights of the primary indicators were 0.268, 0.261, 0.242, and 0.229, respectively, for biological factors, interventions, environmental factors, and social factors. The normalized weights of the top four secondary indicators were 0.122, 0.120, 0.098, and 0.096, respectively, for climatic features, geographical features, sandflies, and dogs. Among the tertiary indicators, the top four normalized comprehensive weights were the population density of sandflies (0.076), topography (0.057), the population density of dogs, including tethering (0.056), and use of bed nets or other protective measures (0.056).

**Conclusions:**

An indicator framework of transmission risk assessment for MT-ZVL was established using the Delphi-entropy weight method. The framework provides a practical tool to evaluate transmission risk in endemic areas.

**Supplementary Information:**

The online version contains supplementary material available at 10.1186/s40249-022-01045-0.

## Background

Visceral leishmaniasis (VL), also known as kala-azar, is a serious disease caused by trypanosomatid protozoans of the genus *Leishmania,* which are transmitted by biting of sandflies from the genera *Phlebotomus* and *Lutzomyia* [1]. If left untreated, VL is fatal in over 95% of cases. VL is one of the most important neglected tropical diseases [2], and is a major global public health problem. *L. donovani* and *L. infantum* are the main *Leishmania* species in China. There are three epidemiological types of VL in China, namely anthroponotic visceral leishmaniasis (AVL), mountain-type zoonotic VL (MT-ZVL), and desert-type zoonotic VL (DT-ZVL), and the main transmitting sandflies for each type of VL are different [3]. *Phlebotomus chinensis* (endophilic species) is the main vector in MT-ZVL endemic areas, including the central and eastern plains, and mountainous and Loess Plateau areas of China [4].

VL was once rampant in rural areas north of the Yangtze River, afflicting more than 600 counties and cities in 16 provincial-level administrative divisions (PLADs), with an estimated number of 530,000 patients in 1951 [5]. Through large-scale prevention and control campaigns, the number of patients decreased yearly [6]. However, with the development of society and management of the environment, more suitable ecological habitats were created for the vector*, Ph. chinensis,* and reservoirs, leading to the re-emergence of MT-ZVL in the hilly areas of China [7]. Since the twenty-first century, the number of MT-ZVL cases reported in central and western China has increased rapidly, and the epidemic region has expanded to more than 60 counties and districts in seven PLADs, including the northern suburbs of Beijing, northern Hebei, western Henan, Shanxi, southern Shaanxi, southern Gansu, and northwestern Sichuan [8, 9]. The proportion of dogs infected with *Leishmania* in endemic areas reached 51.9%, as detected by PCR [10]. A total of 479 MT-ZVL cases were reported in China from 2019 to 2021, and the incidence increased from 0.0010/10,000 in 2019 to 0.0015/10,000 in 2021 [11].

Several studies have been conducted to investigate the risk factors associated with the transmission of the disease based on patterned methods, and found that some meteorological, environmental, and socioeconomic factors could increase the transmission risk of VL [12–16]. The biological activity and size of the sandfly population, as well as that of latent dogs contribute substantially to the dissemination of disease [17]. Regarding biological factors, individual factors, such as the use of bed nets and repellents were considered as influencing factors in several studies [18–20]. However, most studies failed to apply a theoretical and comprehensive framework to identify the specific factors that have the greatest impact on the transmission cycle. It is imperative to monitor and control for such risk factors. Thus, identifying and assessing high-risk factors for the transmission of MT-ZVL is the most important consideration for disease control, including the establishment of public policies, environmental management, treatment of patients, and ensuring public health effectively.

Therefore, it is necessary to develop a comprehensive risk factor analysis tool for MT-ZVL transmission. The Delphi method is an anonymous questionnaire-based method that provides an objectivity and neutrality, as well as use of each expert's knowledge and experience. The method has a certain degree of subjectivity given that it is based on a set of integrated views, and practical and scientific support [21]. The entropy method is an objective method and mainly uses the characteristics of entropy to judge the dispersion degree of each indicator in the framework through the entropy value [22]. Thus, the combination of subjective and objective methods has been used in studies to render results more accurate, reasonable, and effective [23–25]. In this study, the Delphi and entropy methods were applied to establish the multilevel risk factors and comprehensive assessment framework to provide a new basis for the MT-ZVL control in endemic areas.

## Methods

### Establishing a framework for transmission risk

#### Search strategy

The questionnaire was designed using a systematic search approach, which was performed on literature of the risk factors for MT-ZVL. English and Chinese databases, including PubMed, Science Direct, Scopus, Google Scholar, Web of Science, Chinese Biomedical Literature Database (http://www.sinomed.ac.cn/), China National Knowledge Infrastructure (CNKI, http://www.sinomed.ac.cn/), China Science and Technology Journal Database (VIP, http://www.cqvip.com/) and Wanfang (https://www.wanfangdata.com.cn/) were comprehensively searched for published articles on the transmission risk of VL from 2010 to 2022. The search was carried out using the following keywords and terms: “visceral leishmaniasis”, “Kala-azar”, “*Leishmania donovani*”, “*Leishmania infantum*”, “canine visceral leishmaniasis”, “zoonotic visceral leishmaniasis”, “MT-ZVL”, “zoonoses”, “*Phlebotomus*”, “risk factors”, “transmission”, “epidemiology”, “control measures”, alone or in combination with “OR’’ and/or “AND’’ operators. As for grey literature, relevant global or national guidelines for VL were identified from the WHO or other resources.

**Fig. 1:**
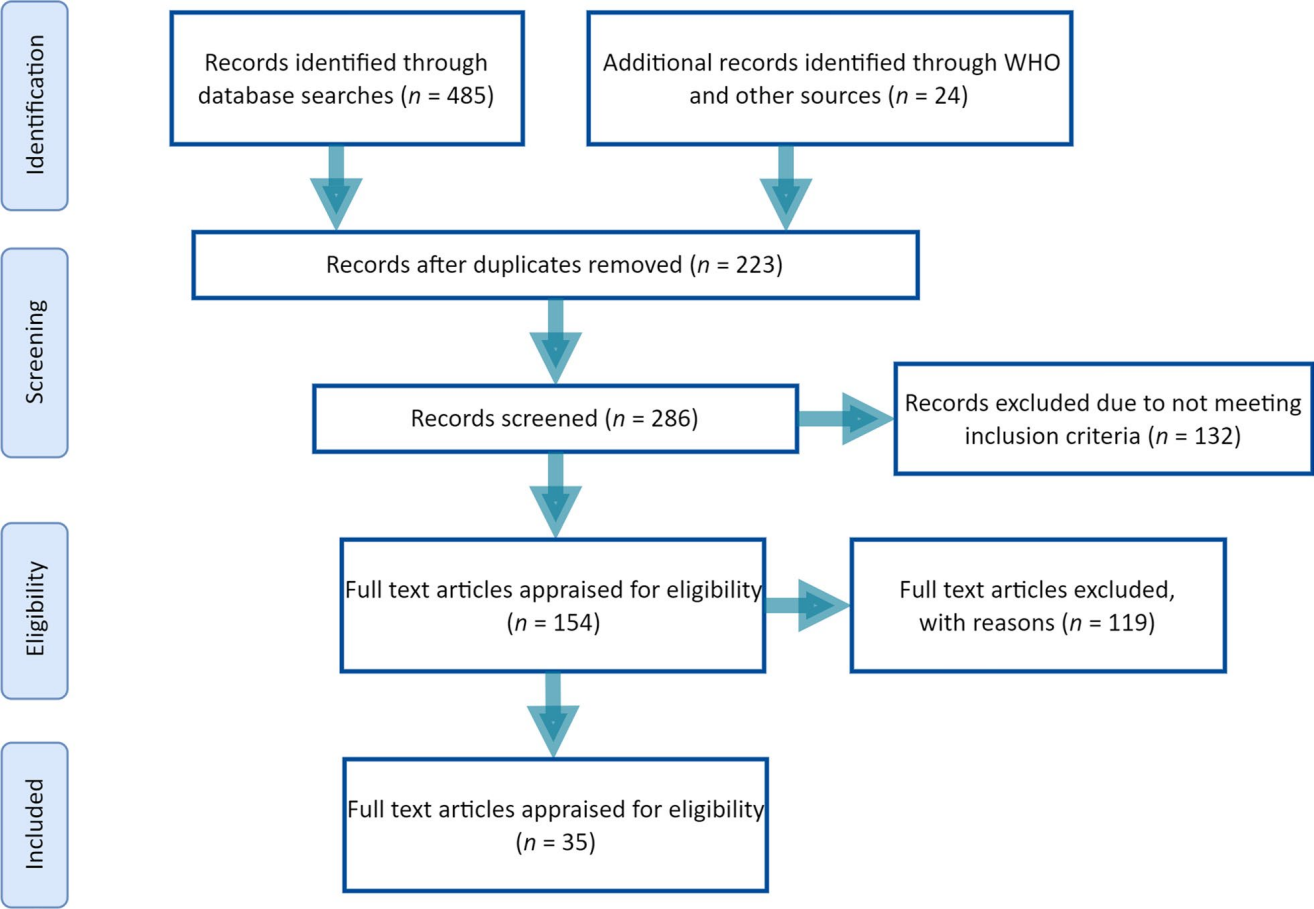
Flowchart of the study selection

Data were extracted from studies with at least one of the following inclusion criteria: studies corresponding to the determination of risk factors of zoonotic visceral leishmaniasis transmission and control strategies. Summaries of articles presented as proceedings at conferences, studies that contained no qualified data, experimental studies, review articles, duplicates, and case reports were excluded (Fig. 1).

#### Criteria for the selection of experts

To ensure the representativeness and authority of the experts, those selected were engaged in the prevention and control of VL from national, provincial, and municipal centers for disease control. The inclusion criteria for experts were ≥ 10 years of work experience in VL research and field prevention and control; familiarity with the pathogenesis and transmission of VL; a bachelor's degree or above; and intermediate or higher professional title. Experts also provided informed consent and volunteered to participate in the study.

#### Design of expert consultation questionnaire

The questionnaire was divided into two parts.

Part I: The core part of the expert inquiry was the importance (*C*_*ij*_) on the transmission risk of each indicator, using a 5-point Likert scale method (5 points: very important, 4 point: important, 3 points: generally important, 2 points: weakly important, and 1 point: not important) based on scientific information, necessity, and operability, and providing qualitative opinions and suggestions.

Part II: Basic information, including general information of experts (age, gender, post, educational background, etc.); familiarity (*C*_*s*_): whether the expert was familiar with the listed indicators and understood the meaning. The highest score was 1, and the higher the score, the greater the familiarity; judgment basis (*C*_*a*_): Based on the expert's judgment, the degree of influence is divided into large, medium, and small. As shown in Table 1, the judgment basis was based on the degree of influence.

**Table 1:** Judgment based on the degree of influence

Judgment basis	Degree of influence		
	Large	Medium	Small
Work experience	0.5	0.4	0.3
Theoretical analysis	0.3	0.2	0.1
References	0.1	0.1	0.1
Intuitive selection	0.1	0.1	0.1

### Calculation of the indicator framework

#### The Delphi method

Two rounds of expert consultations were carried out, and the experts scored the importance (*Cij*), familiarity (*Cs*) and judgment basis (*Ca*) of each indicator of the framework. Experts also provided suggestions for modification and supplementation of the indicators.

After the first round of consultation was complete, the indicator framework was adjusted according to the expert’s scores and suggestions. The second round of expert consultation was then conducted to establish the transmission risk assessment framework. Details on the calculation process are described below:

1. Indicator evaluation score: With the collected data, the assessment criteria were used to assess the indicator framework from those selected experts. An assessment criterion consisted of four parts: (a) positive coefficient: questionnaire response rate; (b) authority coefficient (*C*_*r*_), determined by the judgment basis coefficient (*C*_*a*_) and familiarity coefficient (*C*_*s*_) of the expert. The formula is *C*_*r*_ = (*C*_*a*_ + *C*_*s*_)/2, and the larger *C*_*r*_ value indicates a higher degree of authority of the expert on the content of the consultation; (c) coordination coefficient (Kendall's *W*), the *W* value and its significance test (*χ*^*2*^ test) reflects the degree of dispersion of the expert consultation. *W* is in the range of 0–1. The larger the *W* with a significant *χ*^*2*^ test value was, the better the coordination would be; (d) coefficient of variation (CV), where CV = standard deviation/mean value. The smaller the coefficient of variation, the more unanimous the opinion of experts.
  Then, the weighted importance score (${C}_{ij}^{\prime}$) of each indicator was calculated as follows: ${C}_{ij}^{\prime}= {C}_{ij}\times {C}_{r(ij)}$, which is the product of the importance score and the authority coefficient.
2. Indicator screening: In combination with the suggestions of the experts, the indicator with a weighted importance score, as well scientific information, necessity, and operability score ≥ 3.00 and CV ≤ 40% was retained. Conversely, the weighted importance score < 3.00 or CV > 40% was deleted in the first round. The exclusion criteria for the second round were the weighted importance score < 3.00 or CV > 35%. The criteria for additional indicators should meet 1/3 of the experts suggested, including the indicator.
3. Delphi weight calculation: After the indicators were optimized, the weighted importance score of the primary indicator was first normalized as ${W}_{1,j}$. Subsequently, all of the secondary and tertiary indicators were normalized as ${W}_{2,j},{W}_{3,j}$, respectively. The Delphi normalized weight of the secondary indicators was calculated as ${W}_{d,j=}{W}_{1,j}\times {W}_{2,j}$. Finally, the weight was calculated by continuous multiplication of each tertiary indicator ${W}_{d=}{W}_{1,j}\times {W}_{2,j}\times {W}_{3,j}$, which was the final Delphi weight of each indicator.

#### The entropy method

The entropy weight method is an objective method to determine each indicator’s weight based on the uncertainty contained within each indicator for the whole framework. The concept of entropy is well suited to measuring the relative strength of comparison criterion to represent the average intrinsic information involved in the decision. This method largely avoids the defects of the subjective assignment method on the weight calculation for each indicator, and a greater value indicates a greater incidence for the assessed indicator within the overall evaluation [26]. The entropy weight calculation is as follows:

1. Dimensionless processing: Under the assumption that the indicator framework for transmission risk was assessed through *m* indicators and *n* samples, the original data matrix *X* = ${{(x}_{ij})}_{m\times n}$ was standardized according to the following equation:
  1 $${{x}_{ij}}^{{\prime}+}= \frac{{{x}_{ij}-\mathrm{min}{x}_{i}}}{{\mathrm{max}{x}_{i}-\mathrm{min}{x}_{i}}} {{x}_{ij}}^{{\prime}-}= \frac{{\mathrm{max}{x}_{i}{-x}_{ij}}}{{\mathrm{max}{x}_{i}-\mathrm{min}{x}_{i}}}$$
  where ${{x}_{ij}}^{{\prime}+}$ and ${{x}_{ij}}^{{\prime}-}$ are the positive and negative indicators, respectively; and ${{x}_{ij}}^{\prime}$ is the standardized value for *j*th indicator for the *i*th sample for *i* = 1, 2, …, n and *j* = 1, 2, …, m.
2. (Calculate) The indicator proportion, ${P}_{ij}$, was calculated for each *i* object under each *j* indicator, as follows;
  2 $${P}_{ij}= \frac{{{x}_{ij}}^{\prime}}{{\sum }_{i=1}^{n}{{x}_{ij}}^{\prime}}$$
3. (Calculate) According to the definition of information/entropy [27], ${E}_{j}$ was calculated for the *j*th indicator according to (3).
  3 $${E}_{j}=-k\sum_{i=1}^{n}{p}_{ij}\mathrm{ln}({p}_{ij})$$
  where $k=1/\mathit{ln}(n)$ and ${E}_{j}$ ≥ 0. The difference coefficient, ${G}_{j}$, is calculated as:
  4 $${G}_{j}=\frac{1-{E}_{j}}{m-{E}_{e}}$$
  where ${E}_{e}=\sum_{j=1}^{m}{E}_{j}$; 0 ≤ ${G}_{j}$≤ 1; $\sum_{j=1}^{m}{G}_{j}=1$; a greater value means higher determinacy of the overall evaluation and a smaller entropy.
4. (Calculate) The entropy weight of each indicator, ${W}_{j}$:
  5 $${W}_{j}=\frac{{G}_{j}}{{\sum }_{j=1}^{m}{G}_{j}}$$

### Comprehensive weight

Delphi, analytical hierarchy process (AHP), least square, and binomial coefficient methods were subjective weighting methods. Objective weighting methods mainly include the entropy weight method, principal component analysis method, variance and mean square deviation method, and multi-objective planning method. The Delphi method is highly subjective. Therefore, the combination of subjective and objective methods to jointly establish the weight, complement each other's strengths and weaknesses, respect expert opinions, reflect the objectivity of the data, and reduce the “polarization” effect of different evaluation methods, which can all improve the scientific information of the indicator.

Considering the importance of the indicators and the degree of difference, the weight coefficients obtained from the above two methods were combined by multiplication to obtain the comprehensive weight, ${W}_{c}$ [25]. If there were some indicators with equal scores, the entropy redundancy degree, ${G}_{j}$, was 0 and the comprehensive weight was the Delphi weight (${W}_{a}$= $\sum {W}_{d},{G}_{j}=0$).

6 $${W}_{c}=\left\{\begin{array}{l}{W}_{d},{G}_{j}=0\\ \frac{\left(1-{W}_{a}\right)\times \left({G}_{j}*{W}_{d}\right)}{{\sum }_{j=1}^{m}\left({G}_{j}*{W}_{d}\right)},{G}_{j}\ne 0\end{array}\right.$$

### Statistical analysis

Excel 2020 software (Microsoft Corporation, Redmond, USA) was used to input the expert consultation results. R1.2 software (R Foundation for Statistical Computing, Vienna, Austria) was used to calculate the authority coefficient, weighted importance score, and CV of each indicator. SPSS 26.0 software (IBM Corporation, Armonk, USA) was used to perform Kendall’s *W* test on the weighted importance score at all levels of indicators. The test level was *α* = 0.05.

## Results

### Basic information of experts

The study conducting an indicator framework for the transmission risk of MT-ZVL applied a structured questionnaire to 28 experts during August, 2021. The questionnaire was distributed by a specially-assigned person and the experts were required to reply within one week. All experts were from the Centers for Disease Prevention and Control, and their basic details are provided in Table 2.

**Table 2:** Descriptive statistics of the experts

Subject	Option	Quantity	Proportion (%)
Gender	Male	16	57.2
	Female	12	42.8
Age	Under 40 years old	8	28.5
	40–50 years old	8	28.5
	50–60 years old	10	35.8
	More than 60 years old	2	7.2
Nationality	Chinese	28	100.0
Title	Senior	10	35.7
	Deputy Senior	8	28.6
	Middle	10	35.7
Highest degree	Master and above	11	39.3
	Undergraduate	17	60.7
Years engaged in position	10–20 years	14	50.0
	21–30 years	8	28.5
	More than 31 years	6	21.5

### Expert positive coefficient and authority coefficient

During the first round of this study, two experts refused to participate in the consultation; thus, 30 questionnaires were delivered with 28 valid questionnaires returned. The questionnaire recovery rate was 93.3% and half of the experts made recommendations. In the second round, 28 questionnaires were delivered with 28 valid questionnaires returned. Thus, the positive coefficient of experts was 100.0%, which highlighted that the experts were highly motivated. The authority coefficients of the experts in the two rounds were 0.82 and 0.83, respectively.

### The degree of coordination of expert opinions

The degree of dispersion of the expert consultation was expressed by the coordination coefficient (Kendall’s *W*), *χ*^*2*^ test, and CV. The Kendall’s *W* value of the tertiary indicator in the first round was 0.277 (*χ*^*2*^ = 294.582, *P* < 0.05), and CV ranged from 8% to 45%. In the second round, the *W* value was 0.187 (*χ*^*2*^ = 125.659, *P* < 0.05) and CV ranged from 14% to 34%. A high degree of recognition was demonstrated by the experts, and the outcome was satisfactory. The coordination coefficients for all levels are shown in Table 3.

**Table 3:** Results of two rounds of expert consultation on coordination degree

(Number of) consultations	Indicator hierarchy	Number of indicators	Kendall’s *W*	*χ*^*2*^ value	*P*-value
Round 1	Primary indicators	4	0.252	21.150	< 0.05
	Secondary indicators	12	0.250	76.899	< 0.05
	Tertiary indicators	39	0.277	294.582	< 0.05
Round 2	Primary indicators	4	0.272	22.838	< 0.05
	Secondary indicators	11	0.233	71.886	< 0.05
	Tertiary indicators	25	0.187	125.659	< 0.05

### Deletion and modification of indicators

Through a literature review and expert consultations, an initial transmission risk assessment framework was established that included four primary indicators, 12 secondary indicators and 39 tertiary indicators (Additional file 1). Considering that the contents of some indicators were duplicated, after the first round, one secondary indicator and nine tertiary indicators were deleted, 12 indicators were merged into five, two indicators were modified, and two items were added. Finally, a framework containing 25 tertiary indicators in four dimensions was established (Additional file 1).

### Comprehensive weights based on the Delphi and entropy weight methods

**Fig. 2:**
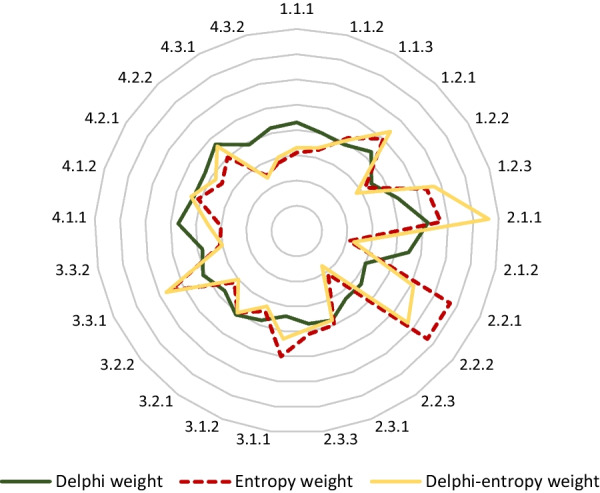
A comparison of the weight coefficient based on the Delphi and entropy weight method

After two rounds of expert consultation, it was finally determined that the framework for the risk assessment of MT-ZVL included four primary indicators, 11 secondary indicators, and 25 tertiary indicators (Additional file 2). The degree of expert’s opinions was expressed with the weighted importance of the indicator and the normalized weight. A larger the score and weight indicated a higher importance for the indicator. The results of expert consultations showed that the weighted importance score of each indicator averaged 3.115–4.322. The normalized weight of the primary indicators based on the Delphi method, ranked from largest to smallest, were biological factors (0.268), interventions (0.261), environmental factors (0.242), and social factors (0.229). The top four Delphi normalized weights of the secondary indicators were climatic features (0.122), geographical features (0.120), sandflies (0.098), and dogs (0.096). The top four tertiary indicators based on the Delphi and entropy weight methods were the density of the sandflies (0.076), the topography (0.057), the population density of dogs, including tethering (0.056), and use of bed nets and other protective measures (0.056). The specific contents are shown in Tables 4, 5, 6 and a comparison obtained by the three weighting methods of the tertiary indicators is shown in Fig. 2.

**Table 4:** Results of the primary indicators of the risk assessment of mountain-type zoonotic visceral leishmaniasis

Indicator code	Primary indicators	Weighted importance score	Coefficient of variation (%)	Normalized weight
1	Environmental factors	3.839 ± 0.743	19	0.242
2	Biological factors	4.251 ± 0.556	13	0.268
3	Social factors	3.633 ± 0.828	22	0.229
4	Interventions	4.126 ± 0.570	13	0.261

**Table 5:** Results of secondary indicators of the risk assessment of mountain-type zoonotic visceral leishmaniasis

Indicator code	Secondary indicators	Weighted importance score	Coefficient of variation (%)	Normalized Delphi weight ${W}_{2,j}$
1.1	Climatic features	3.691 ± 0.911	24	0.122
1.2	Geographical features	3.633 ± 0.943	25	0.120
2.1	Sand flies	4.208 ± 0.634	14	0.098
2.2	Dogs	4.143 ± 0.624	14	0.096
2.3	Livestock	3.194 ± 1.222	34	0.074
3.1	Demographic characteristics	3.345 ± 1.084	31	0.072
3.2	Housing environment	3.616 ± 0.986	26	0.078
3.3	Lifestyle	3.692 ± 0.901	23	0.079
4.1	Reservoirs	4.090 ± 0.615	14	0.090
4.2	Vector	4.065 ± 0.763	18	0.090
4.3	Susceptible population	3.689 ± 1.061	27	0.081

**Table 6:** Results of tertiary indicators of the risk assessment of MT-ZVL

Indicator code	Tertiary indicators	Weighted importance score	Coefficient of variation (%)	Delphi weight ${W}_{d}$	Entropy weight ${W}_{j}$	Comprehensive weights ${W}_{c}$
1	Environmental factors					
1.1	Climatic features					
1.1.1	Monthly/seasonally/annually average temperature	3.622 ± 0.935	26	0.043	0.031	0.034
1.1.2	Monthly/seasonally/annually average precipitation	3.425 ± 0.978	29	0.040	0.033	0.035
1.1.3	Relative humidity	3.324 ± 0.994	30	0.039	0.042	0.042
1.2	Geographical features					
1.2.1	Altitude, latitude, and longitude	3.855 ± 1.019	26	0.043	0.050	0.054
1.2.2	Soil type (sand/silt/clay)	3.115 ± 1.069	34	0.035	0.032	0.028
1.2.3	Topography (plains, mountains, hills, etc.)	3.775 ± 1.019	27	0.042	0.054	0.057
2	Biological factors					
2.1	Sand flies					
2.1.1	Population density	4.322 ± 0.615	14	0.052	0.057	0.076
2.1.2	Natural infection rate	3.707 ± 0.949	26	0.045	0.021	0.023
2.2	Dogs					
2.2.1	Age structure	3.527 ± 1.043	30	0.030	0.067	0.051
2.2.2	Population density including tethering	3.923 ± 0.858	22	0.033	0.067	0.056
2.2.3	Stray dogs and the infected dogs around the house	3.925 ± 0.840	21	0.033	0.021	0.017
2.3	Livestock					
2.3.1	Density of livestock raised	3.340 ± 0.968	29	0.038	0.040	0.038
2.3.2	Whether livestock are free-range	3.197 ± 1.086	34	0.037	0.041	0.039
3	Social factors					
3.1	Demographic characteristics					
3.1.1	Population density	3.140 ± 1.676	34	0.034	0.050	0.043
3.1.2	Age, gender, education level, etc.	3.494 ± 1.047	30	0.038	0.034	0.032
3.2	Housing environment					
3.2.1	Building materials (dirt/brick/tile/concrete)	3.627 ± 0.893	25	0.041	0.040	0.040
3.2.2	Vacant space near the house	3.265 ± 1.110	34	0.037	0.032	0.030
3.3	Lifestyle					
3.3.1	Use of bed nets and other protective measures	3.909 ± 0.750	19	0.041	0.055	0.056
3.3.2	Whether to sleep outdoors	3.607 ± 1.069	30	0.038	0.031	0.030
4	Interventions					
4.1	Reservoirs					
4.1.1	Strengthen the screening and management of dogs	3.970 ± 0.811	20	0.047	0.030	0.035
4.1.2	Dogs are regularly sprayed with insecticides or wear insecticide-impregnated collars	3.676 ± 0.834	23	0.043	0.041	0.044
4.2	Vector					
4.2.1	Regular spraying of insecticides	3.853 ± 0.865	22	0.043	0.035	0.038
4.2.2	Regularly monitor the density of sandflies	4.194 ± 0.794	19	0.047	0.040	0.046
4.3	Susceptible population					
4.3.1	Screening and treatment of villagers with VL	3.945 ± 1.110	28	0.039	0.025	0.025
4.3.2	Awareness rate of VL	4.273 ± 0.643	15	0.042	0.028	0.031

## Discussion

MT-ZVL is considered a canid zoonosis in which sandflies become infected primarily by feeding on the skin of canids, and humans are the final host of the parasites. The control of *Leishmania* infections in the domestic dog population is fundamental in preventing the spread of MT-ZVL between dogs and humans. MT-ZVL has been widely rampant in 10 PLADs in China, including Gansu, Qinghai, Ningxia, Sichuan, Shaanxi, Shanxi, Henan, Hebei, Liaoning, and Beijing [28]. Since the 1960s, MT-ZVL has been controlled through intensive intervention measures to eliminate infectious sources and control sandflies [5]. Nevertheless, natural foci still existed in the mountainous regions in central and western China. As the development of society and the improvement of the ecological environment progresses, MT-ZVL has re-emerged and the endemic areas have been extended over the past few decades [29]. Although some studies have been conducted on the risk factors of MT-ZVL transmission nationally and internationally [30, 31], there is still a lack of scientific and systematic transmission risk assessment indicators.

In this study, a three-level indicator framework for assessing the transmission risk of MT-ZVL was established, which consisted of four primary indicators, 11 secondary indicators, and 25 tertiary indicators. Among the tertiary indicators, the population density of sandflies provided the largest weight, followed by topography, the population density of dogs and tethered dog (tethering), and the use of bed nets and other protective measures, thus, suggesting that the population density of sandflies was the most critical indicator for the risk assessment of MT-ZVL transmission. The rapid resurgence of the MT-ZVL epidemic was closely related to the increase in the population density of sandflies [32], which was consistent with the surveillance results of MT-ZVL in China in recent years. For example, the density of sandflies in Yangquan City in 2021 was as high as 103 sandflies/per human and per hour, as determine by the human baiting method. This density was much higher than that in other MT-ZVL endemic counties. In the same year, a total of 108 MT-ZVL cases were reported in Yangquan City, accounting for 48% (108/224) of MT-ZVL cases reported in China, with an incidence of 0.77/10,000. Yangquan City was also the highest risk area for MT-ZVL in the country [9]. In 2016, MT-ZVL re-emerged in Linzhou in Henan province, where VL had been eliminated for more thirty years [33, 34]. A recent study indicated that environmental (i.e., changes in grasslands/forests), meteorological (i.e., temperature and relative humidity), and socioeconomic (i.e., population density) factors contributed to the recurrence of VL in central China [15], and vector monitoring results showed that the local sandfly density was at a historically high level. In addition, two VL outbreaks occurred in Jiashi County, in Xinjiang Uygur Autonomous Region in 2008 and 2014, respectively, when the population density of sandflies was recorded at a historically high level [35]. Thus, the above surveillance results indicate that the population density of sandflies is an important indicator in risk assessments of MT-ZVL.

Additionally, topography was also considered an important indicator of MT-ZVL transmission risk. Historically, MT-ZVL was mainly distributed in hilly settings of Gansu, Sichuan, Shanxi, Shaanxi, western Henan, and northern Hebei, Qinghai, Ningxia, Liaoning, and the suburbs of Beijing [36]. Such areas were extensions of the Loess Plateau, which provides a suitable habitat for wild host reservoirs and sandflies to maintain the MT-ZVL transmission cycle [16, 37]. Thus, MT-ZVL was closely related to topography and the persistence of the natural habitat of MT-ZVL makes it difficult to prevent the transmission of MT-ZVL in such areas [38]. In recent years, with increases in global warming, the gradual improvement of the natural ecological environment, coupled with the implementation of ecological protection policies such as returning farmland to forests in China, the population density and distribution of wild host reservoirs and sandflies have gradually been restored, the infection rate of dogs has increased, and MT-ZVL has reemerged in previously-endemic counties [39, 40]. Surveillance studies showed that MT-ZVL re-emergence occurred in areas with hilly topographies. Such findings are consistent with the results of our study [8, 41].

The population density of dogs, including tethering, is an important indicator of the risk assessment of MT-ZVL transmission. Dogs are the main host reservoirs of MT-ZVL in China and increases in dog population densities create more favorable reservoirs and higher transmission risks. Previous study also shown that the elimination of dogs in endemic areas dramatically reduced the human VL cases, confirming the infected dogs were the major source of the human infection [10]. In the 2000s, with the number of dogs added significantly, the incidence of MT-ZVL increased rapidly in Jiuzhaigou County, Sichuan province. However, when intervention measures such as dog culling and management were implemented, the incidence declined quickly [42, 43]. In addition, the vector, *Ph. chinensis*, has a small activity radius of usually no more than 300 m [28]. However, free-range style of dogs led to increases in dogs' activities ranges, and increases in the risk of disease transmission. Furthermore, use of bed nets and other protective measures were also crucial indicators for risk assessment. Studies have shown that use of bed nets and indoor insecticides have effectively reduced the risk of human exposure to sandflies and significantly reduced the risk of infection [44]. However, the wild habitat of sandflies in MT-ZVL endemic areas reduced the protective effectiveness of protective measures such as bed nets [27]. Theoretically, the infection rate of dogs and sandflies are important indicators of *Leishmania* transmission and MT-ZVL risk, but the weight value of these indicators in this study were low, which may be due to the difficulty or low operability of detecting the infection rate in sandflies and asymptomatic dogs [45].

The selection of experts was a crucial factor affecting the quality of the Delphi-entropy weight method [46]. To improve the quality of the consultation, all the experts selected had been engaged in VL prevention and control work with over 10 years of field experience. A total of 64.3% (18/28) of the experts held titles of deputy senior or above, and 39.3% (11/28) had a master's degree or above. The valid response rate of the two rounds of expert consultation were above 90%, indicating that the enthusiasm of experts was high [47]. Additionally, a total of 39 opinions were put forward in the two rounds of consultation, indicating that experts had a high degree of attention and support for this study. A high authority coefficient of 0.82 and 0.83, respectively, in each round ensured the authority and reliability of the results. After two rounds of consultation, the importance score of all indicators was 3.115–4.322 points, the CV was 14–34%, and the Kendall’s *W* at 0.187. Compared with the first round, the CV was smaller, suggesting that the degree of fluctuation of expert opinions was small, the degree of coordination was improved, and experts' opinions tended to be consistent. This study not only provides a reasonable, scientifically supported indicator framework for the evaluation of MT-ZVL risk but also found several key indicators by calculating the comprehensive weight. The conclusions of this research may help policymakers to develop guidelines for an effective evaluation method of MT-ZVL risk that can be further validated in different endemic areas. This study may also assist official organizations to identify potential risk factors to prevent the spread of the disease, as well as for the integration and rationalization of resources to ultimately improve the monitoring system in China. Limited by the research conditions, this indicator framework may have some deficiencies. It may be limited by the number of experts consulted, resulting in too few indicators or unbalanced weight coefficients. Due to different backgrounds and experiences of experts, and the meaning of second-round questionnaires are not well explained, so it is difficult to obtain a higher Kendall’s *W* score. Additionally, all experts were from China and further research on a broader national range will enrich the results presented in this study.

## Conclusions

The re-emergence of MT-ZVL has become a serious public health concern. In this study, a risk assessment indicator framework of MT-ZVL was constructed using Delphi-entropy weight method for the first time in China, which consisted of four primary indicators, 11 secondary indicators, and 25 tertiary indicators. Among these indicators, the density of the sandflies, the topography, the population density of dogs and using of bed net were the most critical indicators. The results of this study indicated that the framework can be used to formulate strategies and develop targeted interventions for “vectors-reservoirs-humans” aimed at reducing risk for MT-ZVL control.

## Supplementary Information

**Additional file 1: Table S1.** The indicators for the two rounds of the Delphi consultation.

**Additional file 2: Table S2.** First round of primary indicators of the risk assessment of MT-ZVL.

## Data Availability

Data generated or analyzed during this study are included in this published article and its additional information files.
