# Supplementary material for: Establishment of an indicator framework for the transmission risk of the mountain-type zoonotic visceral leishmaniasis based on the Delphi-entropy weight method
Source: Infect Dis Poverty. 2022 Dec 8;11:122. doi: 10.1186/s40249-022-01045-0 (PMC9730582; doi:10.1186/s40249-022-01045-0)
Supplement: Supplementary file 1 — Additional file 1: Table S1. The indicators for the two rounds of the Delphi consultation. [file 40249_2022_1045_MOESM1_ESM.docx]

**Additional file 1:**

**Table 1.**  The indicators for the two rounds of the Delphi consultation

| The indicators for the first round of the Delphi method | The indicators for the second round of the Delphi method | Modification |
| --- | --- | --- |
| ***Primary* indicators**  1 Environmental factors | 1 Environmental factors |  |
| 2 Biological factors | 2 Biological factors |  |
| 3 Social factors | 3 Social factors |  |
| 4 Interventions | 4 Interventions |  |
| ***Secondary* indicators**  1.1 Climatic features | 1.1 Climatic features |  |
| 1.2 Geographical features | 1.2 Geographical features |  |
| 2.1 Sand flies | 2.1 Sand flies |  |
| 2.2 Dogs | 2.2 Dogs |  |
| 2.3 Livestock | 2.3 Livestock |  |
| 3.1 Demographic characteristics | 3.1 Demographic characteristics |  |
| 3.2 Economic factors | **/** | **Deleted** |
| 3.3 Housing environment | 3.2 Housing environment |  |
| 3.4 Lifestyle | 3.3 Lifestyle |  |
| 4.1 Reservoirs | 4.1 Reservoirs |  |
| 4.2 Vector | 4.2 Vector |  |
| 4.3 Susceptible population | 4.3 Susceptible population |  |
| ***Tertiary* indicators**  1.1.1 Annually average temperature | 1.1.1 Monthly/seasonally/annually average temperature |  |
| 1.1.2 Annually average precipitation | 1.1.2 Monthly/seasonally/annually average precipitation |  |
| 1.1.3 Relative humidity | 1.1.3 Relative humidity |  |
| 1.2.1 Altitude, latitude, and longitude | 1.2.1 Altitude, latitude, and longitude |  |
| 1.2.2 Soil type (sand/silt/clay) | 1.2.2 Soil type (sand/silt/clay) |  |
| 1.2.3 Vegetation (broadleaf, conifer, etc.) | 1.2.3 topography (plains, mountains, hills, etc.) | **Modified** |
| 2.1.1 Population density | 2.1.1 Population density |  |
| 2.1.2 Natural habitat | 2.1.2 Natural infection rate | **Modified** |
| 2.1.3 Resistance | / | **Deleted** |
| 2.2.1 Age structure | 2.2.1 Age structure |  |
| 2.2.2 Number of dogs | 2.2.2 Population density including tethering | **Modified** |
| 2.2.3 Distance between kennel and living room | / | **Deleted** |
| 2.2.4 Whether the dog is free-range | / | **Merged** |
| 2.2.5 Whether the neighbour have dogs | / | **Merged** |
| 2.2.6 Stray dogs nearby | 2.2.3 Stray dogs and the infected dogs around the house | **Modified** |
| 2.3.1 The number of cattle, sheep, chickens, and ducks | 2.3.1The density of livestock raised | **Modified** |
| 2.3.2 Distance between the livestock pen and the living room | / | **Deleted** |
| 2.3.3 Whether livestock are free-range | 2.3.2 Whether livestock are free-range |  |
| 3.1.1 Population density | 3.1.1 Population density |  |
| 3.1.2 Age, gender, education level, etc. | 3.1.2 Age, gender, education level, etc. |  |
| 3.2.1 Gross domestic product | / | **Deleted** |
| 3.2.2 Night light | / | **Deleted** |
| 3.3.1 Building materials (dirt/brick/tile/concrete) | 3.2.1 Building materials (dirt/brick/tile/concrete). |  |
| 3.3.2 Screen doors and windows installed in the house | / | **Deleted** |
| 3.3.3 Vacant space near the house | 3.2.2 Vacant space near the house |  |
| 3.4.1 The use of bed nets | 3.3.1 The use of bed nets and other protective measures | **Modified** |
| 3.4.2 The use of window screens, mosquito coils, repellents, or insecticides | / | **Merged** |
| 3.4.3 Whether to sleep outdoors | 3.3.2 Whether to sleep outdoors |  |
| 3.4.4 Length of time and scope of outdoor activities | / | **Deleted** |
| 4.1.1 Regular health checks on dogs | 4.1.1 Strengthen the screening and management of dogs | **Modified** |
| 4.1.2 Dogs are regularly sprayed with insecticides | 4.1.2 Dogs are regularly sprayed with insecticides or wear insecticide-impregnated collars | **Modified** |
| 4.1.3 Wear a repellent collar | / | **Merged** |
| 4.2.1 Regular spraying of insecticides | 4.2.1 Regular spraying of insecticides |  |
| 4.2.2 Regularly monitor the density of sandflies | 4.2.2 Regularly monitor the density of sandflies |  |
| 4.3.1 Screening in villages with clinical cases | 4.3.1 screening and treatment of villagers with VL | **Modified** |
| 4.3.2 Diagnosis and treatment training on medical staff | / | **Merged** |
| 4.3.3 Provide medicines | / | **Deleted** |
| 4.3.4 Awareness rate of VL | 4.3.2 Awareness rate of VL |  |
| 4.3.5 Hold VL lectures | / | **Deleted** |
