# Supplementary material for: Establishment of an indicator framework for the transmission risk of the mountain-type zoonotic visceral leishmaniasis based on the Delphi-entropy weight method
Source: Infect Dis Poverty. 2022 Dec 8;11:122. doi: 10.1186/s40249-022-01045-0 (PMC9730582; doi:10.1186/s40249-022-01045-0)
Supplement: Supplementary file 2 — Additional file 2: Table S2. First round of primary indicators of the risk assessment of MT-ZVL. [file 40249_2022_1045_MOESM2_ESM.docx]

**Additional file 2:**

**Table 1.** First round of primary indicators of the risk assessment of MT-ZVL

| Indicator code | Primary Indicators | Weighted importance score | Coefficient of variation | Normalized Delphi weight  $W_{1,j}$ |
| --- | --- | --- | --- | --- |
| 1 | Environmental factors | 3.599 ± 0.661 | 18% | 0.243 |
| 2 | Biological factors | 3.984 ± 0.612 | 15% | 0.269 |
| 3 | Social factors | 3.305 ± 0.788 | 24% | 0.223 |
| 4 | Interventions | 3.905 ± 0.606 | 15% | 0.264 |

**Table 2.** First round of secondary indicators of the risk assessment of MT-ZVL

| Indicator code | Secondary Indicators | Weighted importance score | Coefficient of variation | Normalized Delphi weight  $W_{2,j}$ |
| --- | --- | --- | --- | --- |
| 1.1 | Climatic features | 3.301 ± 0.910 | 28% | 0.121 |
| 1.2 | Geographical features | 3.390 ± 0.773 | 23% | 0.123 |
| 2.1 | Sand flies | 3.980 ± 0.731 | 18% | 0.098 |
| 2.2 | Dogs | 3.976 ± 0.704 | 18% | 0.097 |
| 2.3 | Livestock | 3.017 ± 1.137 | 38% | 0.074 |
| 3.1 | Demographic characteristics | 3.136 ± 1.020 | 33% | 0.053 |
| 3.2 | Economic factors | 2.879 ± 1.029 | 36% | 0.049 |
| 3.3 | Housing environment | 3.681 ± 0.777 | 21% | 0.063 |
| 3.4 | Lifestyle | 3.407 ± 0.996 | 29% | 0.058 |
| 4.1 | Reservoirs | 3.997 ± 0.606 | 15% | 0.091 |
| 4.2 | Vector | 4.074 ± 0.740 | 18% | 0.092 |
| 4.3 | Susceptible population | 3.586 ± 1.017 | 28% | 0.081 |

**Table 3.** First round of tertiary indicators of the risk assessment of MT-ZVL

| Indicator code | Tertiary Indicators | Weighted importance score | Coefficient of variation | Normalized Delphi weight  $W_{3,j}$ |
| --- | --- | --- | --- | --- |
| 1.1.1 | Annually average temperature | 3.464 ± 0.161 | 25% | 0.041 |
| 1.1.2 | Annually average precipitation | 3.364 ± 0.184 | 29% | 0.040 |
| 1.1.3 | Relative humidity | 3.250 ± 0.181 | 29% | 0.039 |
| 1.2.1 | Altitude, latitude, and longitude | 3.760 ± 0.193 | 27% | 0.049 |
| 1.2.2 | Soil type (sand/silt/clay) | 3.026 ± 0.199 | 35% | 0.039 |
| 1.2.3 | Vegetation (broadleaf, conifer, etc.) | 2.713 ± 0.193 | 38% | 0.035 |
| 2.1.1 | Population density | 4.138 ± 0.141 | 18% | 0.037 |
| 2.1.2 | Natural habitat | 3.949 ± 0.142 | 19% | 0.035 |
| 2.1.3 | Resistance | 2.969 ± 0.211 | 38% | 0.026 |
| 2.2.1 | Age structure | 3.393 ± 0.214 | 33% | 0.015 |
| 2.2.2 | Number of dogs | 3.955 ± 0.143 | 19% | 0.018 |
| 2.2.3 | Distance between kennel and living room | 3.367 ± 0.220 | 35% | 0.015 |
| 2.2.4 | Whether the dog is free-range | 3.718 ± 0.174 | 25% | 0.017 |
| 2.2.5 | Whether the neighbors have dogs | 3.629 ± 0.197 | 29% | 0.016 |
| 2.2.6 | Stray dogs nearby | 3.783 ± 0.169 | 24% | 0.017 |
| 2.3.1 | The number of cattle, sheep, chickens, and ducks | 3.395 ± 0.184 | 29% | 0.025 |
| 2.3.2 | Distance between the livestock pen and the living room | 3.489 ± 0.185 | 28% | 0.026 |
| 2.3.3 | Whether livestock are free-range | 3.150 ± 0.195 | 33% | 0.023 |
| 3.1.1 | Population density | 3.154 ± 0.218 | 34% | 0.025 |
| 3.1.2 | Age, gender, education level, etc. | 3.389 ± 0.188 | 29% | 0.028 |
| 3.2.1 | Gross domestic product | 3.164 ± 0.216 | 36% | 0.027 |
| 3.2.2 | Night light | 2.590 ± 0.219 | 45% | 0.022 |
| 3.3.1 | Building materials (dirt/brick/tile/concrete) | 3.655 ± 0.156 | 23% | 0.021 |
| 3.3.2 | Screen doors and windows installed in the house | 3.833 ± 0.152 | 21% | 0.022 |
| 3.3.3 | Vacant space near the house | 3.405 ± 0.209 | 33% | 0.020 |
| 3.4.1 | The use of bed nets | 3.864 ± 0.146 | 20% | 0.015 |
| 3.4.2 | The use of window screens, mosquito coils, repellents, or insecticides | 3.855 ±0.148 | 20% | 0.015 |
| 3.4.3 | Whether to sleep outdoors | 3.539 ± 0.193 | 29% | 0.014 |
| 3.4.4 | Length of time and scope of outdoor activities | 3.214 ±0.201 | 33% | 0.013 |
| 4.1.1 | Regular health checks on dogs | 3.922 ± 0.146 | 20% | 0.032 |
| 4.1.2 | Dogs are regularly sprayed with insecticides | 3.653 ± 0.138 | 20% | 0.030 |
| 4.1.3 | Wear a repellent collar | 3.473 ± 0.190 | 29% | 0.028 |
| 4.2.1 | Regular spraying of insecticides | 3.749 ± 0.191 | 27% | 0.044 |
| 4.2.2 | Regularly monitor the density of sandflies | 4.130 ± 0.142 | 18% | 0.048 |
| 4.3.1 | Screening in villages with clinical cases | 3.759 ± 0.224 | 31% | 0.014 |
| 4.3.2 | Diagnosis and treatment training on medical staff | 4.491 ± 0.070 | 8% | 0.017 |
| 4.3.3 | Provide medicines | 4.368 ± 0.104 | 13% | 0.017 |
| 4.3.4 | Awareness rate of VL | 4.223 ± 0.116 | 15% | 0.016 |
| 4.3.5 | Hold VL lectures | 4.283 ± 0.117 | 14% | 0.016 |
